# ESMO-MCBS v2.0: Advances, challenges, and perspectives in the assessment of clinical benefit in oncology

**DOI:** 10.1016/j.jhepr.2025.101553

**Published:** 2025-09-24

**Authors:** Ezequiel Mauro, Miquel Serra-Burriel

**Affiliations:** 1 Liver Cancer Translational Research Group - Institut d'Investigacions Biomèdiques, August Pi i Sunyer (IDIBAPS), Liver Unit-Hospital Clínic, Universitat de Barcelona, Barcelona, Catalonia, Spain; 2 Epidemiology, Biostatistics, and Prevention Institute, University of Zurich, Zurich, Switzerland

Cherny NI, Oosting SF, Dafni U, Latino NJ, Galotti M, Zygoura P, *et al.* ESMO-Magnitude of Clinical Benefit Scale version 2.0 (ESMO-MCBS v2.0). Ann Oncol 2025. https://doi.org/10.1016/J.ANNONC.2025.04.006.

Evaluating clinical benefits in oncology trials is critical for every facet of cancer care: from guiding clinical decisions, to informing regulatory approval, or shaping reimbursement.[1], [2], [3] Unlike statistical significance, therapeutic benefit requires a metric that captures whether a new treatment delivers a clinically meaningful improvement in patient outcomes, a distinction that, in an era of increasingly complex treatment landscapes, carries the difference between real innovation and misguided marginal gains. Therefore, a rigorous and standardized framework for benefit assessment is essential to ensure that innovation translates into real-world values.

The European Society for Medical Oncology Magnitude of Clinical Benefit Scale (ESMO-MCBS) is a structured scoring system that translates trial results into a simple letter ranging from A (highest benefit) to C for curative therapies and from 5 (highest) to 1 for non-curative treatments. The scale assigns benefit scores based on predefined thresholds assessing overall survival (OS), disease-free survival, progression-free survival, quality of life (QoL), and treatment toxicity. As an example, Table 1 summarizes the ESMO-MCBS scores for therapies approved in advanced or unresectable hepatocellular carcinoma (HCC), based on phase III trials. In the first-line setting, atezolizumab-bevacizumab and durvalumab-tremelimumab received the highest score of 5, supported by clinically meaningful gains in median OS (5.8 and 2.6 months), favorable hazard ratios (HR 0.66 and 0.78), and improvement in QoL. Durvalumab monotherapy was scored 4, based on non-inferiority (HR 0.86) and lower toxicity. Lenvatinib also met non-inferiority criteria (HR 0.92) but showed no QoL benefit and was classified as having no evaluable clinical benefit. In the second-line setting, cabozantinib (OS gain: 2.2 months; HR 0.76) and regorafenib (OS gain: 2.8 months; HR 0.63) provided modest survival benefits without QoL improvements. Notably, regorafenib was downgraded from score 4 to 3 in ESMO-MCBS v2.0 due to stricter scoring criteria (Table S1). Ramucirumab, restricted to patients with AFP ≥400 ng/ml, showed minimal benefit (OS gain: 1.2 months; HR 0.71) and received the lowest score of 1.

**Table 1:** Summary of ESMO-MCBS scores for approved therapies in advanced HCC.

Therapy	Setting	Median OS gain	HR (95% CI)	QoL impact	ESMO-MCBS score
Atezolizumab-bevacizumab	First-line	5.8 months	0.66 (0.52-0.85)	Delayed deterioration	5 (Form 2a)
Durvalumab-tremelimumab	First-line	2.6 months 3-year OS gain: 10.9% [26% (103/393) of patients in experimental arm evaluable at 3 years]	0.78 (0.67-0.92)	Delayed deterioration	5 (Form 2a)
Durvalumab (non-inferiority)	First-line	2.8 months	0.86 (0.74-1.01)	Delayed deterioration, less toxicity	4 (Form 2c)
Lenvatinib (non-inferiority)	First-line	1.3 months	0.92 (0.79-1.06)	No QoL or toxicity benefit	NEB (Form 2c)
Sorafenib	First-line	2.8 months	0.69 (0.55-0.87)	No QoL benefit	3 (Form 2a)
Regorafenib	Second-line	2.8 months	0.63 (0.50-0.79)	QoL not qualified for credit	3 (Form 2a, v2.0)
Cabozantinib	Second-line	2.2 months	0.76 (0.63-0.92)	QoL not qualified for credit	3 (Form 2a)
Ramucirumab (AFP ≥400 ng/ml)	Second-line	1.2 months	0.71 (0.53-0.95)	No QoL benefit	1 (Form 2a)

ESMO-MCBS, European Society for Medical Oncology magnitude of clinical benefit scale; HR, hazard ratio; OS, overall survival; QoL, quality of life.

The newly released version 2.0 introduces several pivotal refinements that update the scale’s precision, applicability, and alignment with the contemporary trial methodology. Among these are i) strengthened landmark requirements, analyses of late survival now mandate that at least 20% of randomized patients remain at risk at the prespecified time point, preserving statistical power; ii) clearer toxicity annotations that distinguish acute from persistent adverse events and offer explicit criteria for each, facilitating more nuanced shared decision-making; and iii) the introduction of an intermediate benefit category, which allows trials with meaningful disease-free survival improvements in curative-intent settings to be recognized, even in the absence of mature OS data. While this last point is a potential step forward, its operationalization and clinical relevance may require further validation. These updates represent incremental progress and reflect a growing consensus on best methodological practices.^1^ (Fig. 1; Table S1).

**Fig. 1:**
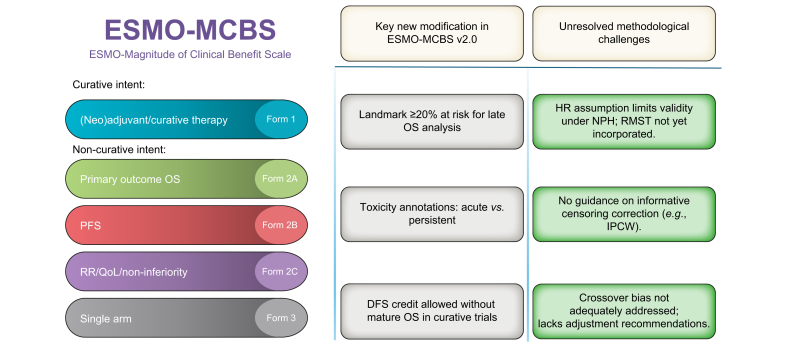
Overview of ESMO-MCBS v2.0 structure, key modifications, and remaining methodological challenges. DFS, disease-free survival; ESMO-MCBS, European Society for Medical Oncology magnitude of clinical benefit scale; HR, hazard ratio; IPCW, inverse-probability-of-censoring weighting; NPH, non-proportional hazards; OS, overall survival; PFS, progression-free survival; QoL, quality of life; RMST, restricted mean survival time; RR, response rate.

Despite these advances, version 2.0 does not fully resolve several methodological challenges that increasingly characterize modern oncology trials.

First, the scale still relies on the assumption of proportional HR, making it inadequate in the presence of non-proportional hazards (NPH).^4^^,^^5^ Under NPH, HR varies over time. This is not a theoretical concern; such patterns are common in immunotherapy trials, including first-line studies in HCC, where delayed, diminishing, or crossing effects are frequent. Accurate assessment in these settings requires mature event data, NPH-sensitive tests (*e.g*. MaxCombo test), and effect measures that do not depend on the proportional hazards (PH) assumption.^6^

In this context, the restricted mean survival time (RMST) has emerged as a key alternative.^7^ RMST provides a straightforward and intuitive estimate of average survival within a clinically defined time horizon, offering an interpretable measure of benefits that does not require PH. It reflects the actual time the average patient gains within the follow-up of the study, making it particularly suitable for informing patient-physician discussions and policy level decisions. Despite its growing adoption in high-impact oncology journals, increasing support from statisticians, and endorsement by the FDA,[6], [8], [9], [10], [11], [19] RMST has not been integrated into ESMO-MCBS v2.0. This omission weakens the ability of the scale to reflect patient-relevant outcomes. Unlike HR, which can be opaque under NPH, RMST offers a transparent metric that supports shared decision-making and better aligns with patient expectations. Additionally, it allows for the translation of drug efficacy into cost-effectiveness analyses in the form of life-years gained, making it a crucial metric for reimbursement and coverage decisions. The HCC setting provides a clear example of the limitations of the current ESMO-MCBS framework. Four pivotal trials (IMbrave050, LEAP-012, HIMALAYA, and CheckMate 9DW) exhibit NPH, highlighting how the absence of alternative methodological approaches in such scenarios may bias benefit assessment.^6^ Moreover, although ESMO-MCBS v2.0 introduces tumor-specific guidance for defining OS data maturity, HCC is not included among the listed malignancies. This omission restricts the applicability of the amendment to HCC trials, where survival dynamics differ markedly from other tumor types, leaving the assessment of maturity subjective and potentially inconsistent.

Second, the scale offers limited guidance on how to handle informative censoring, a well-recognized source of bias in time-to-event endpoints.^12^ When dropout or censoring occurs non-randomly between treatment arms, often due to toxicity or dissatisfaction of trial arm allocation in non-blinded studies, early progression, or differential follow-up, key endpoints such as disease-, progression-, recurrence-free survival can become severely biased.^13^^,^^14^ This issue is particularly relevant in perioperative or curative-intent trials, where the control group may consist of observation alone, and censoring mechanisms differ substantially. Without explicit recommendations for detecting and correcting for informative censoring, such as IPCW (inverse-probability-of-censoring weighting) or sensitivity testing, the derived benefit scores may substantially bias the true clinical effect. Future iterations of the ESMO-MCBS should therefore provide clear, methodologically grounded instructions for detecting, analyzing, and reporting informative censoring to ensure that estimates of therapeutic benefit remain both robust and clinically meaningful.

Third, patient crossover from the control to the experimental arm remains a major methodological challenge that ESMO-MCBS v2.0, addresses only tangentially. In superiority trials, crossover attenuates the observed treatment effect; in non-inferiority designs, it can spuriously reinforce claims of equivalence, distorting OS analyses used as either primary or secondary endpoints.^15^ While crossover is often ethically justified, particularly in advanced disease settings, it must be accompanied by pre-specified adjustment methods, such as RPSFT (rank-preserving structural failure-time models), IPCW, or instrumental variable models, along with transparent sensitivity analyses.^16^

Embedding these requirements into future ESMO-MCBS updates is critical. Failure to do so could lead, for example, to equating the therapeutic benefit of a truly innovative drug with another one with no meaningful efficacy but with a trial plagued with informative censoring.

Comparatively, frameworks from ASCO (the American Society of Clinical Oncology)^17^ and the NCCN (National Comprehensive Cancer Network)^18^ offer distinct perspectives. The ASCO Value Framework and NCCN integrate patient-reported outcomes and symptom management to provide a more holistic view of treatment benefits.^17^

ESMO-MCBS v2.0 marks an important evolution in the structured evaluation of clinical benefit and significantly enhances its alignment with contemporary oncology trials. Nonetheless, future iterations must move beyond the PH paradigm and integrate validated alternatives, such as RMST, NPH-sensitive tests, and trial maturity thresholds for this setting. Equally, explicit guidance on managing informative censoring and patient crossover is essential to avoid biased estimates and to preserve the integrity of survival endpoints. Embedding these refinements would not only enhance methodological robustness, but also ensure that the scale remains clinically meaningful, aligned with patient priorities, and fit for guiding therapeutic decisions, regulatory evaluation, and value-based reimbursement in modern oncology.

## Authors’ contributions

Both authors contributed equally.

## Financial support

The authors did not receive any financial support to produce this manuscript.

## Declaration of generative AI and AI-assisted technologies in the writing process

During the preparation of this work the authors used ChatGPT (OpenAI) to improve grammar and style of the manuscript. After using this ChatGPT, the authors reviewed and edited the content as needed and take full responsibility for the content of the publication.

## Conflict of interest

EM received travel funding from Roche. MSB: None.

Please refer to the accompanying ICMJE disclosure forms for further details.
