# Supplementary material for: ESMO-MCBS v2.0: Advances, challenges, and perspectives in the assessment of clinical benefit in oncology
Source: JHEP Rep. 2025 Sep 24;7(10):101553. doi: 10.1016/j.jhepr.2025.101553 (PMC12541619; doi:10.1016/j.jhepr.2025.101553)
Supplement: Multimedia component 1 [file mmc1.pdf]

ESMO-MCBS v2.0: Advances, challenges, and perspectives in the assessment of clinical benefit in oncology

Ezequiel Mauro, Miquel Serra-Burriel

Table of content

Table S1……………………………………………………………………………………2

Table S1. Detailed summary of modifications in ESMO-MCBS v2.0

Nº.	Identified Issue (ESMO-MCBS v1.1)	Modification (ESMO-MCBS v2.0)	Rationale (Key Examples)	Type of Modification
1	Lack of explicit method to estimate median survival if not reached in experimental arm.	Explicit rule: median in experimental arm estimated by dividing control median survival by the HR.	CheckMate 214 (renal cancer): Nivolumab+Ipilimumab vs. Sunitinib; experimental median OS not reached initially.	Technical
2	Overly lenient thresholds for HR and absolute DFS gains.	Stricter HR criteria (≤0.65 for highest scores) and mandatory absolute gain constraints.	APHINITY and ExteNET trials: High scores despite small absolute gains (2.5–2.8%).	Nuanced (restrictive)
3	No acknowledgment of DFS benefit when OS gain	DFS gains are credited, but score reduced by one level if OS is	Breast and colon adjuvant studies previously marked as “no evaluable benefit”	Nuanced (clinical relevance)

	was not significant.	ultimately not significant.	despite significant DFS improvement.	
4	No explicit guidance on maturity criteria for OS data.	Clearly defined OS maturity thresholds based on cancer type (e.g., 3–10 years depending on tumor).	Heterogeneous maturity definitions previously caused discrepancies.	Technical
5	Arbitrary (3-year) OS evaluation threshold.	OS credited whenever statistically significant irrespective of specific follow-up period.	NSABP C-03 (colorectal cancer): OS benefit observed before conventional maturity.	Nuanced & Technical
6	No toxicity evaluation in curative-intent therapies.	Introduced annotations (non-penalizing) for acute (AT) and persistent toxicities (PT).	ExteNET: 40% severe diarrhea; Taxanes/Oxaliplatin: persistent neuropathy.	Structural & Technical

7	No form available to score single-arm de-escalation studies.	New form (1b) for single-arm de-escalation trials achieving pre-specified outcomes.	Trastuzumab+paclitaxel regimen (HER2+ breast cancer): excellent 10-year DFS.	Structural
8	Excessive crediting of OS Tail-of-the-Curve (ToC) with few evaluable patients.	Minimum requirement: ≥20% of patients evaluable at ToC timepoints.	RESORCE (regorafenib in HCC): only 5.5% evaluable, now ineligible for ToC credit.	Nuanced (statistical rigor)
9	Overly permissive thresholds for long median OS (≥36 months).	New sub-form for studies with control median OS ≥36 months.	STAMPEDE (prostate cancer): downgraded from 4 to 3 due to modest absolute gain.	Structural
10	Unable to score OS when gain occurs before median OS is reached.	New rule allows scoring significant OS benefits even before median OS is reached.	ARAMIS: significant 3-year OS gain without median OS reached.	Nuanced

11	Lenient thresholds for long PFS in control arms (≥12 months).	New sub-form for studies with control median PFS ≥12 months.	Breast, prostate, lung cancer trials now properly stratified.	Structural
12	Excessive ToC credit for PFS with few evaluable patients.	ToC credit requires ≥20% evaluable unless gain ≥25%.	KEYNOTE-002 lost ToC credit; CROWN retained due to ≥25% PFS gain.	Nuanced (statistical rigor)
13	Inadequate toxicity criteria for penalizing adverse events.	Stricter criteria: ≥10% discontinuation or hospitalization, ≥2% fatal AEs, etc.	Olaparib, niraparib, pazopanib studies now subject to appropriate penalties.	Nuanced (clinical relevance)
