# Supplementary material for: ESMO-MCBS v2.0: Advances, challenges, and perspectives in the assessment of clinical benefit in oncology
Source: JHEP Rep. 2025 Sep 24;7(10):101553. doi: 10.1016/j.jhepr.2025.101553 (PMC12541619; doi:10.1016/j.jhepr.2025.101553)
Supplement: Multimedia component 2 [file mmc2.docx]

**Supplementary Table 1. Detailed summary of modifications in ESMO-MCBS v2.0**

| **N^o^.** | **Identified Issue (ESMO-MCBS v1.1)** | **Modification (ESMO-MCBS v2.0)** | **Rationale**  **(Key Examples)** | **Type of Modification** |
| --- | --- | --- | --- | --- |
| 1 | Lack of explicit method to estimate median survival if not reached in experimental arm. | Explicit rule: median in experimental arm estimated by dividing control median survival by the HR. | CheckMate 214 (renal cancer): Nivolumab+Ipilimumab vs. Sunitinib; experimental median OS not reached initially. | Technical |
| 2 | Overly lenient thresholds for HR and absolute DFS gains. | Stricter HR criteria (≤0.65 for highest scores) and mandatory absolute gain constraints. | APHINITY and ExteNET trials: High scores despite small absolute gains (2.5–2.8%). | Nuanced (restrictive) |
| 3 | No acknowledgment of DFS benefit when OS gain was not significant. | DFS gains are credited, but score reduced by one level if OS is ultimately not significant. | Breast and colon adjuvant studies previously marked as “no evaluable benefit” despite significant DFS improvement. | Nuanced (clinical relevance) |
| 4 | No explicit guidance on maturity criteria for OS data. | Clearly defined OS maturity thresholds based on cancer type (e.g., 3–10 years depending on tumor). | Heterogeneous maturity definitions previously caused discrepancies. | Technical |
| 5 | Arbitrary (3-year) OS evaluation threshold. | OS credited whenever statistically significant irrespective of specific follow-up period. | NSABP C-03 (colorectal cancer): OS benefit observed before conventional maturity. | Nuanced & Technical |
| 6 | No toxicity evaluation in curative-intent therapies. | Introduced annotations (non-penalizing) for acute (AT) and persistent toxicities (PT). | ExteNET: 40% severe diarrhea; Taxanes/Oxaliplatin: persistent neuropathy. | Structural & Technical |
| 7 | No form available to score single-arm de-escalation studies. | New form (1b) for single-arm de-escalation trials achieving pre-specified outcomes. | Trastuzumab+paclitaxel regimen (HER2+ breast cancer): excellent 10-year DFS. | Structural |
| 8 | Excessive crediting of OS Tail-of-the-Curve (ToC) with few evaluable patients. | Minimum requirement: ≥20% of patients evaluable at ToC timepoints. | RESORCE (regorafenib in HCC): only 5.5% evaluable, now ineligible for ToC credit. | Nuanced (statistical rigor) |
| 9 | Overly permissive thresholds for long median OS (≥36 months). | New sub-form for studies with control median OS ≥36 months. | STAMPEDE (prostate cancer): downgraded from 4 to 3 due to modest absolute gain. | Structural |
| 10 | Unable to score OS when gain occurs before median OS is reached. | New rule allows scoring significant OS benefits even before median OS is reached. | ARAMIS: significant 3-year OS gain without median OS reached. | Nuanced |
| 11 | Lenient thresholds for long PFS in control arms (≥12 months). | New sub-form for studies with control median PFS ≥12 months. | Breast, prostate, lung cancer trials now properly stratified. | Structural |
| 12 | Excessive ToC credit for PFS with few evaluable patients. | ToC credit requires ≥20% evaluable unless gain ≥25%. | KEYNOTE-002 lost ToC credit; CROWN retained due to ≥25% PFS gain. | Nuanced (statistical rigor) |
| 13 | Inadequate toxicity criteria for penalizing adverse events. | Stricter criteria: ≥10% discontinuation or hospitalization, ≥2% fatal AEs, etc. | Olaparib, niraparib, pazopanib studies now subject to appropriate penalties. | Nuanced (clinical relevance) |
