# Supplementary material for: ESMO-MCBS v2.0: Advances, challenges, and perspectives in the assessment of clinical benefit in oncology
Source: JHEP Rep. 2025 Sep 24;7(10):101553. doi: 10.1016/j.jhepr.2025.101553 (PMC12541619; doi:10.1016/j.jhepr.2025.101553)
Supplement: Multimedia component 3 [file mmc3.pdf]

ESMO-MCBS v2.0: Advances, challenges, and perspectives in the assessment of clinical benefit in oncology

Cherny NI, Oosting SF, Dafni U, Latino NJ, Galotti M, Zygoura P, *et al.* ESMO-Magnitude of Clinical Benefit Scale version 2.0 (ESMO-MCBS v2.0). Ann Oncol 2025. https://doi.org/10.1016/J.ANNONC.2025.04.006.

Evaluating clinical benefits in oncology trials is critical for every facet of cancer care: from guiding clinical decisions, to informing regulatory approval, or shaping reimbursement.[1–3] Unlike statistical significance, therapeutic benefit requires a metric that captures whether a new treatment delivers a clinically meaningful improvement in patient outcomes, a distinction that, in an era of increasingly complex treatment landscapes, carries the difference between real innovation and misguided marginal gains. Therefore, a rigorous and standardized framework for benefit assessment is essential to ensure that innovation translates into real-world values.

The European Society for Medical Oncology Magnitude of Clinical Benefit Scale (ESMO-MCBS) is a structured scoring system that translates trial results into a simple letter ranging from A (highest benefit) to C for curative therapies and from 5 (highest) to 1 for non-curative treatments. The scale assigns benefit scores based on predefined thresholds assessing overall survival (OS), disease-free survival, progression-free survival, quality of life (QoL), and treatment toxicity. As an example, Table 1 summarizes the ESMO-MCBS scores for therapies approved in advanced or unresectable hepatocellular carcinoma (HCC), based on phase III trials. In the first-line setting, atezolizumab-bevacizumab and durvalumab-tremelimumab received the highest score of 5, supported by clinically meaningful gains in median OS (5.8 and 2.6 months), favorable hazard ratios (HR 0.66 and 0.78), and improvement in QoL. Durvalumab monotherapy was scored 4, based on non-inferiority (HR 0.86) and lower toxicity. Lenvatinib also met non-inferiority criteria (HR 0.92) but showed no QoL benefit and was classified as having no evaluable clinical benefit. In the second-line setting, cabozantinib (OS gain: 2.2 months; HR 0.76) and regorafenib (OS gain: 2.8 months; HR 0.63) provided modest survival benefits without QoL improvements. Notably, regorafenib was downgraded from score 4 to 3 in ESMO-MCBS v2.0 due to stricter scoring criteria (Table S1). Ramucirumab, restricted to patients with AFP ≥400 ng/ml, showed minimal benefit (OS gain: 1.2 months; HR 0.71) and received the lowest score of 1.

The newly released version 2.0 introduces several pivotal refinements that update the scale's precision, applicability, and alignment with the contemporary trial methodology. Among these are i) strengthened landmark requirements, analyses of late survival now mandate that at least 20% of randomized patients remain at risk at the prespecified time point, preserving statistical power; ii) clearer toxicity annotations that distinguish acute from persistent adverse events and offer explicit criteria for each, facilitating more nuanced shared decision-making; and iii) the introduction of an intermediate benefit category, which allows trials with meaningful disease-free survival improvements in curative-intent settings to be recognized, even in the absence of mature OS data. While this last point is a potential step forward, its operationalization and clinical relevance may require further validation. These updates represent incremental progress and reflect a growing consensus on best methodological practices.[1] (Fig. 1; Table S1).

Despite these advances, version 2.0 does not fully resolve several methodological challenges that increasingly characterize modern oncology trials.

First, the scale still relies on the assumption of proportional HR, making it inadequate in the presence of non-proportional hazards (NPH).[4,5] Under NPH, HR varies over time. This is not a theoretical concern; such patterns are common in immunotherapy trials, including first-line studies in HCC, where delayed, diminishing, or crossing effects are frequent. Accurate assessment in these settings requires mature event data, NPH-sensitive tests (*e.g.* MaxCombo test), and effect measures that do not depend on the proportional hazards (PH) assumption.[6]

In this context, the restricted mean survival time (RMST) has emerged as a key alternative.[7] RMST provides a straightforward and intuitive estimate of average survival within a clinically defined time horizon, offering an interpretable measure of benefits that does not require PH. It reflects the actual time the average patient gains within the follow-up of the study, making it particularly suitable for informing patient-physician discussions and policy level decisions. Despite its growing adoption in high-impact oncology journals, increasing support from statisticians, and endorsement by the FDA,[6,8–11,19] RMST has not been integrated into ESMO-MCBS v2.0. This omission weakens the ability of the scale to reflect patient-relevant outcomes. Unlike HR, which can be opaque under NPH, RMST offers a transparent metric that supports shared decision-making and better aligns with patient expectations. Additionally, it allows for the translation of drug efficacy into cost-effectiveness analyses in the form of life-years gained, making it a crucial metric for reimbursement and coverage decisions. The HCC setting provides a clear example of the limitations of the current ESMO-MCBS framework. Four pivotal trials (IMbrave050, LEAP-012,

HIMALAYA, and CheckMate 9DW) exhibit NPH, highlighting how the absence of alternative methodological approaches in such scenarios may bias benefit assessment.[6] Moreover, although ESMO-MCBS v2.0 introduces tumor-specific guidance for defining OS data maturity, HCC is not included among the listed malignancies. This omission restricts the applicability of the amendment to HCC trials, where survival dynamics differ markedly from other tumor types, leaving the assessment of maturity subjective and potentially inconsistent.

Second, the scale offers limited guidance on how to handle informative censoring, a well-recognized source of bias in time-to-event endpoints.[12] When dropout or censoring occurs non-randomly between treatment arms, often due to toxicity or dissatisfaction of trial arm allocation in non-blinded studies, early progression, or differential follow-up, key endpoints such as disease-, progression-, recurrence-free survival can become severely biased.[13,14] This issue is particularly relevant in perioperative or curative-intent trials, where the control group may consist of observation alone, and censoring mechanisms differ substantially. Without explicit recommendations for detecting and correcting for informative censoring, such as IPCW (inverse-probability-of-censoring weighting) or sensitivity testing, the derived benefit scores may substantially bias the true clinical effect. Future iterations of the ESMO-MCBS should therefore provide clear, methodologically grounded instructions for detecting, analyzing, and reporting informative censoring to ensure that estimates of therapeutic benefit remain both robust and clinically meaningful.

Third, patient crossover from the control to the experimental arm remains a major methodological challenge that ESMO-MCBS v2.0, addresses only tangentially. In superiority trials, crossover attenuates the observed treatment effect; in non-inferiority designs, it can spuriously reinforce claims of equivalence, distorting OS analyses used as either primary or

Table 1. Summary of ESMO-MCBS scores for approved therapies in advanced HCC.

Therapy	Setting	Median OS gain	HR (95% CI)	QoL impact	ESMO-MCBS score
Atezolizumab-bevacizumab	First-line	5.8 months	0.66 (0.52-0.85)	Delayed deterioration	5 (Form 2a)
Durvalumab-tremelimumab	First-line	2.6 months<br>3-year OS gain: 10.9% [26% (103/393) of patients in experimental arm evaluable at 3 years]	0.78 (0.67-0.92)	Delayed deterioration	5 (Form 2a)
Durvalumab (non-inferiority)	First-line	2.8 months	0.86 (0.74-1.01)	Delayed deterioration, less toxicity	4 (Form 2c)
Lenvatinib (non-inferiority)	First-line	1.3 months	0.92 (0.79-1.06)	No QoL or toxicity benefit	NEB (Form 2c)
Sorafenib	First-line	2.8 months	0.69 (0.55-0.87)	No QoL benefit	3 (Form 2a)
Regorafenib	Second-line	2.8 months	0.63 (0.50-0.79)	QoL not qualified for credit	3 (Form 2a, v2.0)
Cabozantinib	Second-line	2.2 months	0.76 (0.63-0.92)	QoL not qualified for credit	3 (Form 2a)
Ramucirumab (AFP ≥400 ng/ml)	Second-line	1.2 months	0.71 (0.53-0.95)	No QoL benefit	1 (Form 2a)

ESMO-MCBS, European Society for Medical Oncology magnitude of clinical benefit scale; HR, hazard ratio; OS, overall survival; QoL, quality of life.

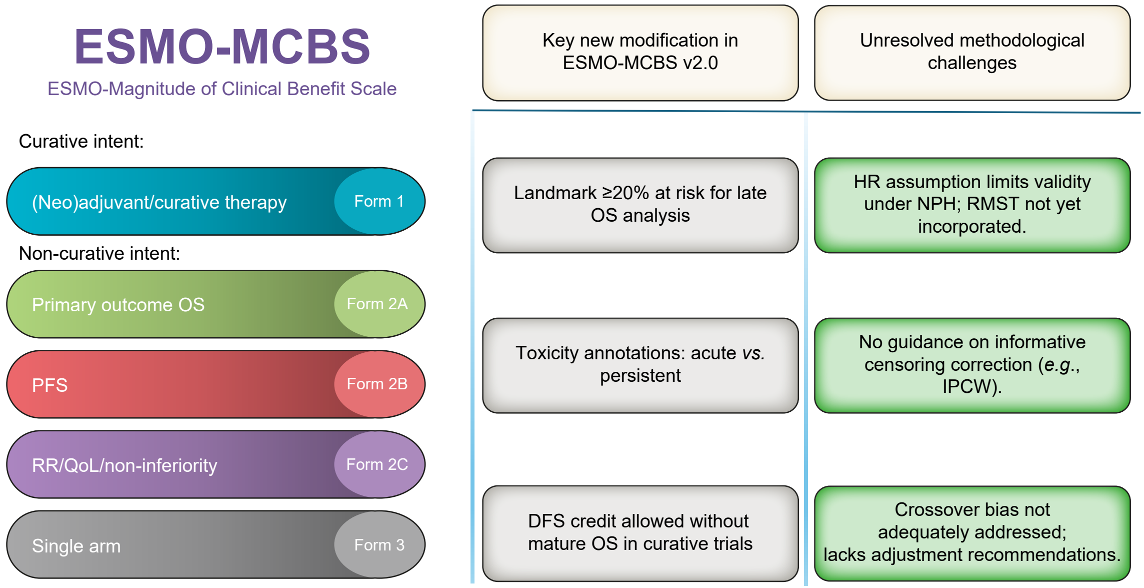

Fig. 1. Overview of ESMO-MCBS v2.0 structure, key modifications, and remaining methodological challenges. DFS, disease-free survival; ESMO-MCBS, European Society for Medical Oncology magnitude of clinical benefit scale; HR, hazard ratio; IPCW, inverse-probability-of-censoring weighting; NPH, non-proportional hazards; OS, overall survival; PFS, progression-free survival; QoL, quality of life; RMST, restricted mean survival time; RR, response rate.

secondary endpoints.[15] While crossover is often ethically justified, particularly in advanced disease settings, it must be accompanied by pre-specified adjustment methods, such as RPSFT (rank-preserving structural failure-time models), IPCW, or instrumental variable models, along with transparent sensitivity analyses.[16]

Embedding these requirements into future ESMO-MCBS updates is critical. Failure to do so could lead, for example, to equating the therapeutic benefit of a truly innovative drug with another one with no meaningful efficacy but with a trial plagued with informative censoring.

Comparatively, frameworks from ASCO (the American Society of Clinical Oncology)[17] and the NCCN (National Comprehensive Cancer Network)[18] offer distinct perspectives. The ASCO Value Framework and NCCN integrate patient-reported outcomes and symptom management to provide a more holistic view of treatment benefits.[17]

ESMO-MCBS v2.0 marks an important evolution in the structured evaluation of clinical benefit and significantly enhances its alignment with contemporary oncology trials. Nonetheless, future iterations must move beyond the PH paradigm and integrate validated alternatives, such as RMST, NPH-sensitive tests, and trial maturity thresholds for this setting. Equally, explicit guidance on managing informative censoring and patient crossover is essential to avoid biased estimates and to preserve the integrity of survival endpoints. Embedding these refinements would not only enhance methodological robustness, but also ensure that the scale remains clinically meaningful, aligned with patient priorities, and fit for guiding therapeutic decisions, regulatory evaluation, and value-based reimbursement in modern oncology.

Ezequiel Mauro[1]
Miquel Serra-Burriel[2,*]
[1]*Liver Cancer Translational Research Group - Institut d'Investigacions Biomèdiques, August Pi i Sunyer (IDIBAPS), Liver Unit-Hospital Clínic, Universitat de Barcelona, Barcelona, Catalonia, Spain*
[2]*Epidemiology, Biostatistics, and Prevention Institute, University of Zurich, Zurich, Switzerland*

Financial support

The authors did not receive any financial support to produce this manuscript.

Conflict of interest

EM received travel funding from Roche. MSB: None.
Please refer to the accompanying ICMJE disclosure forms for further details.

Authors' contributions

Both authors contributed equally.

Declaration of generative AI and AI-assisted technologies in the writing process

During the preparation of this work the authors used ChatGPT (OpenAI) to improve grammar and style of the manuscript. After using this ChatGPT, the authors reviewed and edited the content as needed and take full responsibility for the content of the publication.

Supplementary data

Supplementary data to this article can be found online at https://doi.org/10.1016/j.jhepr.2025.101553.

References

Author names in bold designate shared co-first authorship

[1] Cherny NI, Oosting SF, Dafni U, et al. ESMO-magnitude of clinical benefit scale version 2.0 (ESMO-MCBS v2.0). Ann Oncol 2025. https://doi.org/10.1016/J.ANNONC.2025.04.006.
[2] Kanavos P, Visintin E, Angelis A. Use of the ESMO-Magnitude of Clinical Benefit Scale to guide HTA recommendations on coverage and reimbursement for cancer medicines: a retrospective analysis. Lancet Oncol 2024;25:1644–1654. https://doi.org/10.1016/S1470-2045(24)00505-9.
[3] Vokinger KN, Hwang TJ, Grischott T, et al. Prices and clinical benefit of cancer drugs in the USA and Europe: a cost–benefit analysis. Lancet Oncol 2020;21:664–670. https://doi.org/10.1016/S1470-2045(20)30139-X.
[4] Alexander BM, Schoenfeld JD, Trippa L. Hazards of hazard ratios - deviations from model assumptions in immunotherapy. N Engl J Med 2018;378:1158–1159. https://doi.org/10.1056/NEJMC1716612.
[5] Rahman R, Fell G, Ventz S, et al. Deviation from the proportional hazards assumption in randomized phase 3 clinical trials in oncology: prevalence, associated factors, and implications. Clin Cancer Res 2019;25:6339–6345. https://doi.org/10.1158/1078-0432.CCR-18-3999.
[6] Mauro E, de Castro T, Zeitlhoefler M, et al. OS-060-YI Phase III trials in hepatocellular carcinoma: IMbrave 050 and strategies to address non-proportional hazards. J Hepatol 2025;82:S45. https://doi.org/10.1016/S0168-8278(25)00375-7.
[7] Dehbi HM, Royston P, Hackshaw A. Life expectancy difference and life expectancy ratio: two measures of treatment effects in randomised trials with non-proportional hazards. BMJ (Online) 2017;357. https://doi.org/10.1136/bmj.j2250.
[8] European Medicines Agency. ICH E9 (R1) addendum on estimands and sensitivity analysis in clinical trials to the guideline on statistical principles for clinical trials - step 2b. EMA 2017;44.
[9] Blank CU, Lucas MW, Scolyer RA, et al. Neoadjuvant Nivolumab and ipilimumab in Resectable stage III Melanoma. New Engl J Med 2024. https://doi.org/10.1056/NEJMOA2402604/SUPPL_FILE/NEJMOA2402604_DATA-SHARING.PDF.
[10] Schmid P, Cortes J, Dent R, et al. Overall survival with Pembrolizumab in early-stage triple-negative breast cancer. N Engl J Med 2024;391:1981–1991. https://doi.org/10.1056/NEJMOA2409932.
[11] **Yau T**, **Galle PR**, Decaens T, et al. Nivolumab plus ipilimumab versus lenvatinib or sorafenib as first-line treatment for unresectable hepatocellular carcinoma (CheckMate 9DW): an open-label, randomised, phase 3 trial. Lancet 2025. https://doi.org/10.1016/S0140-6736(25)00403-9.
[12] Templeton AJ, Amir E, Tannock IF. Informative censoring — a neglected cause of bias in oncology trials. Nat Rev Clin Oncol 2020;17:327–328. https://doi.org/10.1038/S41571-020-0368-0.
[13] Locher L, Serra-Burriel M, Trapani D, et al. Why effect sizes are systematically larger for progression-free survival than overall survival in cancer drug trials: prognostic scores as a way forward. Eur J Cancer 2024;213:115106. https://doi.org/10.1016/j.ejca.2024.115106.
[14] Lesan V, Olivier T, Prasad V. Progression-free survival estimates are shaped by specific censoring rules: implications for PFS as an endpoint in cancer randomized trials. Eur J Cancer 2024;202. https://doi.org/10.1016/j.ejca.2024.114022.
[15] Haslam A, Prasad V. When is crossover desirable in cancer drug trials and when is it problematic? Ann Oncol 2018;29:1079. https://doi.org/10.1093/ANNONC/MDY116.
[16] Watkins C, Kleine E, Miranda M, et al. Further practical guidance on adjusting time-to-event outcomes for treatment switching. Pharm Stat 2025;24. https://doi.org/10.1002/PST.70019.
[17] Schnipper LE, Davidson NE, Wollins DS, et al. Updating the American society of clinical oncology value framework: revisions and reflections in response to comments received. J Clin Oncol 2016;34:2925–2933. https://doi.org/10.1200/JCO.2016.68.2518.
[18] Carlson RW, Jonasch E. NCCN evidence blocks. JNCCN J Natl Compr Cancer Netw 2016;14:616–619. https://doi.org/10.6004/JNCCN.2016.0177.
[19] Approaches to Assessment of Overall Survival in Oncology Clinical Trials | FDA n.d. https://www.fda.gov/regulatory-information/search-fda-guidancedocuments/approaches-assessment-overall-survival-oncology-clinical-trials.

Journal of Hepatology, Volume ■

Supplemental information

ESMO-MCBS v2.0: Advances, challenges, and perspectives in the assessment of clinical benefit in oncology

Ezequiel Mauro and Miquel Serra-Burriel

ESMO-MCBS v2.0: Advances, challenges, and perspectives in the assessment of clinical benefit in oncology

Ezequiel Mauro, Miquel Serra-Burriel

Table of content

Table S1……………………………………………………………………………………2

Table S1. Detailed summary of modifications in ESMO-MCBS v2.0

Nº.	Identified Issue (ESMO-MCBS v1.1)	Modification (ESMO-MCBS v2.0)	Rationale (Key Examples)	Type of Modification
1	Lack of explicit method to estimate median survival if not reached in experimental arm.	Explicit rule: median in experimental arm estimated by dividing control median survival by the HR.	CheckMate 214 (renal cancer): Nivolumab+Ipilimumab vs. Sunitinib; experimental median OS not reached initially.	Technical
2	Overly lenient thresholds for HR and absolute DFS gains.	Stricter HR criteria (≤0.65 for highest scores) and mandatory absolute gain constraints.	APHINITY and ExteNET trials: High scores despite small absolute gains (2.5–2.8%).	Nuanced (restrictive)
3	No acknowledgment of DFS benefit when OS gain	DFS gains are credited, but score reduced by one level if OS is	Breast and colon adjuvant studies previously marked as “no evaluable benefit”	Nuanced (clinical relevance)

	was not significant.	ultimately not significant.	despite significant DFS improvement.	
4	No explicit guidance on maturity criteria for OS data.	Clearly defined OS maturity thresholds based on cancer type (e.g., 3–10 years depending on tumor).	Heterogeneous maturity definitions previously caused discrepancies.	Technical
5	Arbitrary (3-year) OS evaluation threshold.	OS credited whenever statistically significant irrespective of specific follow-up period.	NSABP C-03 (colorectal cancer): OS benefit observed before conventional maturity.	Nuanced & Technical
6	No toxicity evaluation in curative-intent therapies.	Introduced annotations (non-penalizing) for acute (AT) and persistent toxicities (PT).	ExteNET: 40% severe diarrhea; Taxanes/Oxaliplatin: persistent neuropathy.	Structural & Technical

7	No form available to score single-arm de-escalation studies.	New form (1b) for single-arm de-escalation trials achieving pre-specified outcomes.	Trastuzumab+paclitaxel regimen (HER2+ breast cancer): excellent 10-year DFS.	Structural
8	Excessive crediting of OS Tail-of-the-Curve (ToC) with few evaluable patients.	Minimum requirement: ≥20% of patients evaluable at ToC timepoints.	RESORCE (regorafenib in HCC): only 5.5% evaluable, now ineligible for ToC credit.	Nuanced (statistical rigor)
9	Overly permissive thresholds for long median OS (≥36 months).	New sub-form for studies with control median OS ≥36 months.	STAMPEDE (prostate cancer): downgraded from 4 to 3 due to modest absolute gain.	Structural
10	Unable to score OS when gain occurs before median OS is reached.	New rule allows scoring significant OS benefits even before median OS is reached.	ARAMIS: significant 3-year OS gain without median OS reached.	Nuanced

11	Lenient thresholds for long PFS in control arms (≥12 months).	New sub-form for studies with control median PFS ≥12 months.	Breast, prostate, lung cancer trials now properly stratified.	Structural
12	Excessive ToC credit for PFS with few evaluable patients.	ToC credit requires ≥20% evaluable unless gain ≥25%.	KEYNOTE-002 lost ToC credit; CROWN retained due to ≥25% PFS gain.	Nuanced (statistical rigor)
13	Inadequate toxicity criteria for penalizing adverse events.	Stricter criteria: ≥10% discontinuation or hospitalization, ≥2% fatal AEs, etc.	Olaparib, niraparib, pazopanib studies now subject to appropriate penalties.	Nuanced (clinical relevance)
